# The Wanderer: Acute Abdomen Secondary to a Wandering Spleen

**DOI:** 10.1155/crra/6697671

**Published:** 2025-06-10

**Authors:** Toni Lombardo, Rishi R. Shah, Joseph Mazzie

**Affiliations:** ^1^New York Institute of Technology College of Osteopathic Medicine, Old Westbury, New York, USA; ^2^Nova Southeastern University, Fort Lauderdale, Florida, USA

## Abstract

Wandering spleen is a rare condition resulting from the absence or laxity of peritoneal attachments, allowing the spleen to migrate within the abdominal cavity. While some patients may remain asymptomatic, others can develop life-threatening complications such as splenic torsion, infarction, and bowel obstruction. This case report highlights one of the complications of wandering spleen and the importance of timely diagnosis and intervention.

## 1. Introduction

Wandering spleen is a rare disease due to the lack of peritoneal attachments to the spleen or laxity of those attachments [1]. While many patients may be asymptomatic and the wandering spleen may be an incidental finding, others may present with acute abdominal findings secondary to splenic torsion, infarction, or necrosis. Due to the absence of attachments, the spleen can be found in different locations within the abdomen and the pelvis on imaging, even on the same patient done days apart [2]. Due to the risk of infections and sepsis after splenectomy, early diagnosis and splenopexy are recommended in these patients [3].

This entity was first described in 1854 by Józef Dietl, a Polish physician who described three separate case reports of a wandering spleen and believed it was the relaxation, extension, or hypoplasia of the splenic ligaments that caused this condition [4].

## 2. Case Presentation

**Figure 1:**
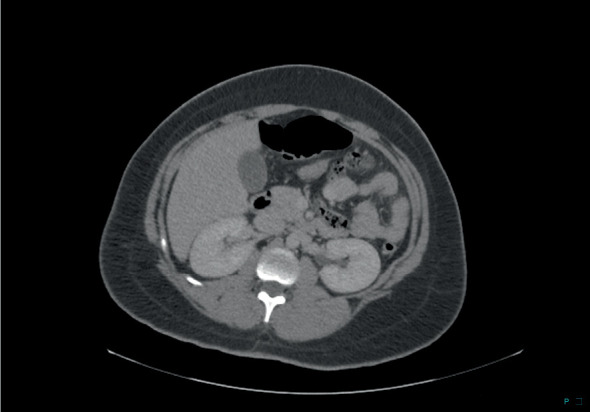
CT image shows an absent spleen in the left upper quadrant.

**Figure 2:**
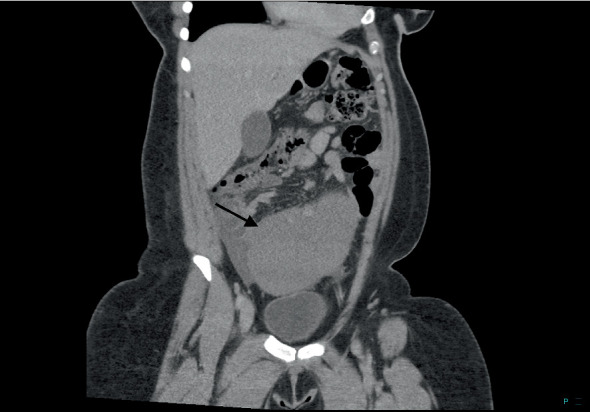
CT scan demonstrated the spleen located above the bladder rather than in the left upper quadrant.

**Figure 3:**
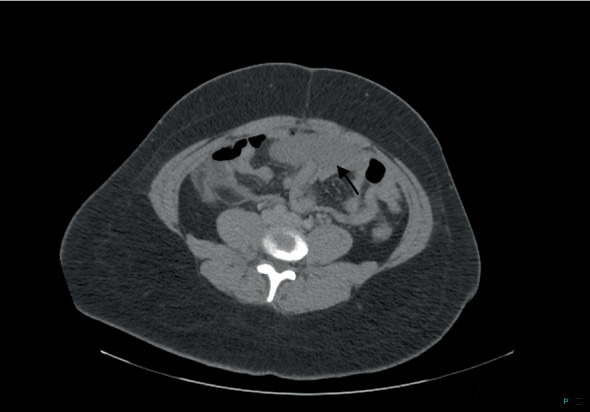
CT image shows a wandering spleen in the lower abdomen indicated by the arrow.

**Figure 4:**
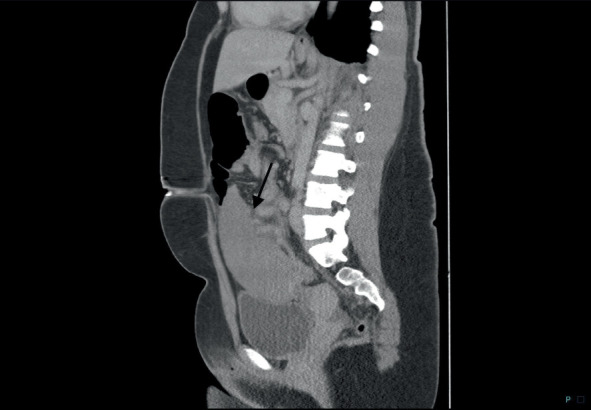
CT image shows an anteriorly located spleen in the lower quadrants of the abdomen indicated by the arrow.

**Figure 5:**
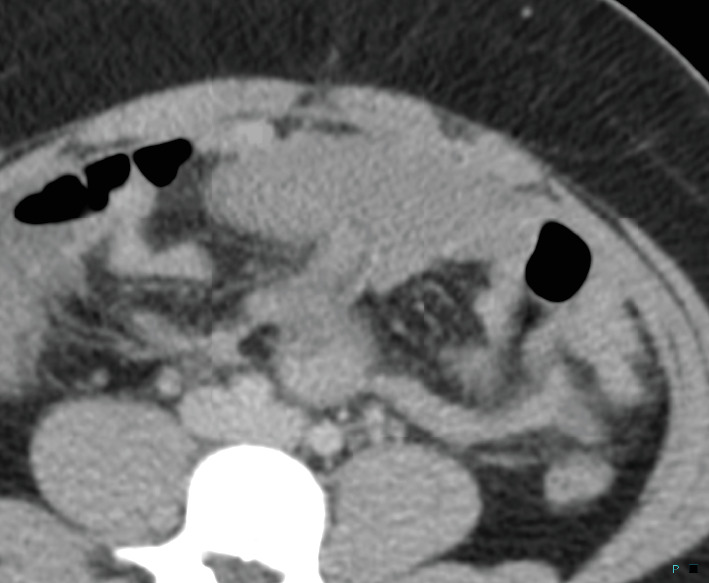
Enlarged image of CT scan demonstrates torsed pedicle of the spleen located in the anterior abdomen.

A 27-year-old female with a past medical history of hypertension and GERD presented to the hospital with worsening abdominal pain. The patient had previously presented to her primary care physician with left upper quadrant pain and heartburn and was prescribed omeprazole. The following day, she described worsening pain which had now spread to her entire abdomen with worsening on the left, and generalized body aches. A CT scan with IV contrast was performed, imaged in the portal venous phase of imaging following 100 mL intravenous contrast. On that study, the spleen was described as absent, and a 13 × 8 cm soft tissue density in the lower left hemiabdomen that was initially described as a possible hematoma (Figures 1 and 2). She was then discharged from the emergency room with a presumed diagnosis of intraperitoneal hematoma, presumed secondary to rupture of a hemorrhagic ovarian cyst, as the severity of the patient's symptoms was not severe enough to require a hospital admission. She, however, returned the following day with worsening pain and an acute abdomen which prevented her from ambulating. At this time, the patient's imaging studies were reviewed again. On further review, the abnormality in the left lower abdomen demonstrated that the density and location of the previously described finding were consistent with a spleen and were now felt to represent a wandering spleen, and tracing the vascular supply and venous drainage to this structure also was consistent with a wandering spleen (Figures 3 and 4). Additionally, on questioning, the patient reported that after a car accident 10 years ago, she was told that her spleen was not in its normal anatomic position; although in the absence of symptoms, surgical intervention was not pursued at that time. The patient went on to undergo an exploratory laparotomy, which confirmed a wandering spleen with complete torsion, thrombosed splenic vein leading to splenomegaly, and pinning of the transverse colon by the splenic pedicle causing bowel obstruction with massive dilation of the proximal colon (Figure 5). An internal hernia with incarceration of small intestines from adhesions resulting in hemorrhagic changes to the involved intestinal walls and mesentery was present, as was an acutely inflamed appendix. The patient was discharged 9 days after splenectomy, appendectomy, and lysis of adhesions with resolution of symptoms.

## 3. Discussion

This case demonstrates a complication associated with wandering spleen, a rare condition that may remain asymptomatic in many patients, as it was in this patient for many years until the acute episode described above. This condition may result in splenic torsion and splenic vein thrombosis, and obstruction by the splenic pedicle can result in symptomatic bowel obstruction and compromise the vascular supply to the bowel and mesentery, resulting in an acute abdomen and the need for emergent surgical intervention.

Although the wandering spleen had been diagnosed 10 years previously, neither the emergency room clinical staff nor the radiologist was aware of this rare condition in this patient at the time of presentation, which may have potentially heightened suspicion and allowed for earlier diagnosis of the etiology of the patient's pain. An overall increased awareness of this rare entity may also have allowed for an earlier diagnosis to have been made.

Early intervention with splenopexy, surgically fixing the spleen in its proper anatomic position, can potentially prevent such complications. Splenopexy is considered in cases where the spleen is seen as viable and done in order to prevent further complications that may threaten that viability or damage to other organs. This procedure may also allow patients to keep a functioning spleen to prevent complications associated with asplenia, such as increased susceptibility to infections and increased risk of sepsis postsplenectomy. Splenopexy is only an option in cases with a viable spleen with no evidence of infarct, thus reserved usually for asymptomatic cases and as a preventative surgical intervention for cases in which the spleen itself is not torsed. It may be performed open or laparoscopic, depending on patient and surgeon preference [5]. Although few cases have been reported in the literature, splenopexy for wandering spleen has good reported outcomes without recurrent symptoms or future complications [6]. In cases where patients are presenting with acute abdomen, as in this case, and abnormal location of the spleen confirmed, exploratory laparotomy and urgent splenectomy are often needed due to torsion and secondary infarction [7].

Wandering spleen makes up only 0.5% of splenectomies and represents a very rare condition generally; however, the risks that can be associated with this condition make early diagnosis and treatment important. When imaging indicates a lack of a spleen in the upper left quadrant, it is important to keep wandering spleen as a possible differential, especially in the setting of an acute abdomen. Both CT and MRI imaging may also provide useful information for the location and viability of the spleen [8].

## 4. Conclusion

Wandering spleen is a rare condition that may remain benign in patients for years and may only present itself as an incidental finding on imaging used for other diagnoses. The spleen may be palpated as a pelvic or abdominal mass on a physical exam. In other cases, such as the one discussed here, the complications associated with a wandering spleen will present in the setting of an acute abdomen. Splenic torsion and vein thrombosis are complications associated with the spleen's mobility and lack of attachments. In this case, the wandering spleen resulted in bowel obstruction and compromised vascular supply to the bowel and mesentery, resulting in an acute abdomen.

## Data Availability

Data sharing is not applicable to this article, as no new data were created or analyzed in this study.
